# Metformin Treatment Potentially Modifies Genetically Driven Metabolite-HbA1c Associations: A Gene–Environment Interaction Mendelian Randomization Study

**DOI:** 10.3390/ph19050780

**Published:** 2026-05-15

**Authors:** Najeha Anwardeen, Aleem Razzaq, Asma A. Elashi, Gaurav Thareja, Ilhame Diboun, Khaled Naja, Karsten Suhre, Mohamed A. Elrayess

**Affiliations:** 1 Biomedical Research Center, QU Health, Qatar University, Doha P.O. Box 2713, Qatar; n.anwardeen@qu.edu.qa (N.A.); aleem.razzaq@qu.edu.qa (A.R.); asma.elashi@qu.edu.qa (A.A.E.); khaled.naja@qu.edu.qa (K.N.); 2 Bioinformatics Core, Weill Cornell Medicine-Qatar, Education City, Doha P.O. Box 24144, Qatarkas2049@qatar-med.cornell.edu (K.S.); 3 Department of Human Genetics, Sidra Medicine, Doha P.O. Box 26999, Qatar; idiboun@sidra.org; 4 Englander Institute for Precision Medicine, Weill Cornell Medicine, New York, NY 10065, USA; 5 College of Medicine, QU Health, Qatar University, Doha P.O. Box 2713, Qatar

**Keywords:** metformin, metabolomics, mendelian randomization, precision medicine

## Abstract

**Introduction/Background**: Metformin is the first-line therapy for type 2 diabetes (T2D); however, a considerable inter-individual variability in glycemic response is observed among patients. This heterogeneity suggests that metformin’s effects depend not only on drug exposure but also on the underlying metabolic and genetic factors. **Methods**: We applied a Gene–Environment interaction Mendelian Randomization (MR-G×E) in a cohort of 2743 individuals to investigate whether genetically influenced metabolite-HbA1c associations differ by metformin use. Metabolites associated with metformin response were used to establish metabolite-specific polygenic risk scores (PRSs) using metabolome-wide association study (mGWAS) variants. Generated PRS were used as genetic instruments within a one-sample, modified two-stage least squares model. An interaction term between PRS and metformin use was included to assess treatment-dependent genetic effects, adjusting for age, sex, body mass index, and genetic ancestry (principal components). **Results**: Metformin use significantly modified genetically influenced associations between 18 metabolites and HbA1c. Positive and negative PRS-metformin interaction effects indicated attenuation, strengthening or reversal of baseline genetic associations under treatment. Several amino acid metabolites, palmitoyl sphingomyelin (d18:1/16:0), and carbohydrate-related metabolite 1,5-anhydroglucitol showed specific patterns under metformin use. Interestingly, several metabolites (creatinine, gamma glutamylcitrulline, N-acetylthreonine, 3-methyl-2-oxovalerate, glycerol-3-phosphate, 1-(1-enyl-palmitoyl)-GPC (P-16:0), 1-(1-enyl-palmitoyl)-2-linoleoyl-GPC (P-16:0/18:2), sphingomyelin (d18:1/22:1, d18:2/22:0, d16:1/24:1), fructose, and methyl-glucopyranoside (alpha + beta)) showed no basal causal association with HbA1c but exhibited significant interaction effect with metformin use, suggesting metabolic association only in the presence of metformin. **Conclusions**: These findings indicate that metformin modifies the genetically influenced metabolite-HbA1c relationships, exhibiting treatment-dependent metabolic effects that are not detectable with standard MR approaches. Incorporating pharmacological context into causal inference provides new insights into the metabolic basis for the variable metformin response and helps inform precision strategies for T2D management.

## 1. Introduction

Type 2 diabetes (T2D) is a heterogeneous metabolic disorder characterized by a considerable inter-individual variability in glycemic control and treatment response [1]. Metformin remains the first-line pharmacotherapy for diabetic individuals; however, a considerable proportion of individuals fail to achieve adequate glycemic improvement despite adhering to the treatment for many years [2]. Therefore, understanding additional factors underlying biological heterogeneity that can affect metformin response is essential for effective treatment of T2D. Recent research has concentrated on type 2 diabetes risk and plasma and serum metabolite profiles [3]. Metabolomics has emerged as a leading methodology for precision medicine for biomarker discovery as well as understanding the underlying molecular mechanism of disease. This includes diabetes diagnosis, prognosis, and management through customized phenotyping and drug-response monitoring [4].

Circulating metabolites, such as amino acids, sugars, lipids, nucleotides, among others, are the byproducts of biological functions, and studying the metabolome aids in understanding an organism’s metabolic processes and physiological status, offering insights into health, illness, and exposure effects, including glycemic control to treatment [5]. Previously, we have shown that metformin response-associated metabolites, such as sphingomyelins, acylcholines, and glutathione metabolites, were elevated in good responders, and poor responders had higher amounts of metabolites from the gut microbiota and glucose metabolism [6].

Additionally, factors affecting responsiveness to metformin therapy include genetic variants associated with plasma membrane amine transporter (PMAT), organic cation transporters (OCT), and multidrug and toxin extrusion (MATE) transporter [7,8], which are the primary determinants of metformin response. Genome-wide association studies (GWAS) have identified numerous variants associated with metabolic traits and glycemic phenotypes [9,10]; however, translating these findings into clinically useful insights remains challenging [11]. Studies combining genomics in conjunction with high-throughput metabolomics data have made it possible to identify several loci for characteristics linked to circulating amino acids, fatty acids, lipids and lipoproteins [12,13,14]. These investigations have shed new light on the biology of human metabolism and directed extensive epidemiological research, including the use of Mendelian Randomization (MR) methods to determine causal correlations [14].

Most of the previous studies investigated genetic variants related to metformin response using the genome-wide approaches along with pharmacogenomics, while MR studies were also conducted to investigate the causal effect of metabolites on glycemic traits. These traditional MR analyses estimate an average causal effect across the population, implicitly assuming that genetic effects are constant and independent of environmental or treatment contexts [15]. However, this assumption may be violated in pharmacological settings where drug exposure can modify biological pathways linking the exposure to the outcome, resulting in effect heterogeneity rather than horizontal pleiotropy [16]. Although the MR-G×Eapproach was developed to overcome such heterogeneity, the implementation of this approach in the context of drug-metabolite, especially metformin, was not investigated before.

In this study, we applied the MR-G×Eapproach to investigate whether genetically influenced variation in circulating metabolites associated with glycemic outcomes is dependent on metformin use. We focused on the metabolites that were previously associated with metformin response as endogenous exposures mediating the causal pathway between genetic predisposition and glycemic control. PRS constructed from metabolite-associated genetic variants identified from metabolome-wide association studies (mGWAS) are used as instruments for these metabolite levels. Metformin use is modeled as an exogenous binary treatment variable and as an effect modifier of genetic influence. The interaction between the metabolite-PRS and metformin use serves as an additional instrument to further identify the treatment-dependent genetic effects on HbA1c.

Incorporating pharmacological context into causal MR analysis, this new approach can reveal metabolic pathways that could be modified by metformin exposure, helping to explain inter-individual variability in glycemic responses and supporting more targeted, metabolite-based strategies for T2D management.

## 2. Results

In this study, a Gene–Environment interaction approach of MR was performed to investigate whether the causal effect of genetically influenced metabolites on HbA1c differs between metformin users and non-metformin users.

A polygenic risk score (PRS) for each metabolite was generated separately using SNPs associated with respective metabolites at *p* < 5 × 10^−6^. These PRS were evaluated in relation to HbA1c while adjusting for age, gender, and BMI. An interaction term between PRS and metformin use (binary) was included to assess treatment-dependent modification of genetic effects. Using this approach, metformin was found to significantly modify the causal association between genetically predicted levels of 18 metabolites and HbA1c when comparing metformin users with non-metformin users.

Among metformin users, several metabolites demonstrated a significant positive PRS-metformin interaction effect, implying that their genetic causal effect on HbA1c is stronger for metformin users compared with non-metformin users. These metabolites included glycine, glutamine, palmitoyl sphingomyelin, 1-ribosyl-imidazole acetate, pyroglutamine, gamma-glutamyl citrulline, 1-enyl-palmitoyl-2-linoleoyl-GPC, pro-hydroxy pro, N-acetyl threonine, perfluorooctanoate, 3-methyl-2-oxovalerate, and fructose. The strongest positive interaction effects were observed for glycine (β = 0.175, *p*-value ≤ 2 × 10^−16^), glutamine (β = 0.123, *p*-value = 1.62 × 10^−8^), and palmitoyl sphingomyelin (d18:1/16:0) (β = 0.118, *p*-value = 5.34 × 10^−7^).

**Figure 1:**
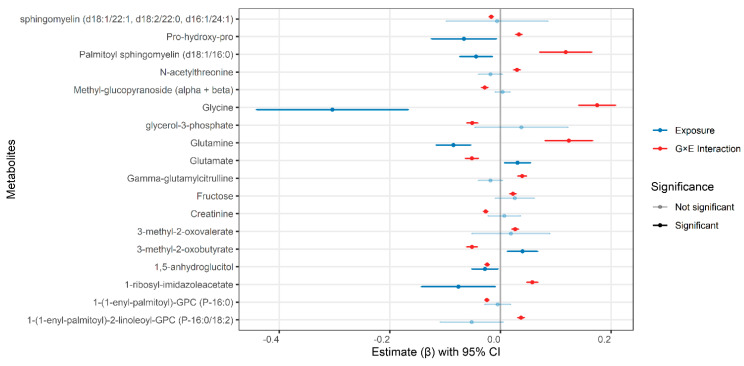
Metformin-modified causal effects of circulating metabolites on HbA1c. Points represent model-based effect estimates (β) from MR analysis, and error bars indicate 95% confidence intervals. Blue points denote the main genetic effect of each metabolite on HbA1c, while red points represent the genotype × metformin interaction effect, reflecting modification of the metabolite-HbA1c association in the presence of metformin use. The positive interaction estimate represents the genetic effect of the metabolite on HbA1c is stronger in metformin users, while a negative interaction estimate represents attenuation or reversal of the genetic effect under metformin treatment. The gray vertical line indicates the null effect (β = 0). Estimates with 95% confidence intervals overlapping zero are shown with reduced opacity and are not statistically significant, whereas fully opaque points indicate significant results.

In contrast, several metabolites showed a significant negative PRS-metformin interaction effect, indicating metformin’s attenuation or reversal of the effect of genetic risk on glycemic control in metformin users. These metabolites included sphingomyelin (d18:1/22:1, d18:2/22:0, d16:1/24:1), 1,5-anhydroglucitol, 1-(1-enyl-palmitoyl)-GPC (P-16:0), creatinine, methyl glucopyranoside (alpha + beta), glycerol-3-phosphate, 3-methyl-2-oxobutyrate, and glutamate. The strongest negative interaction effects were observed for the glutamate (β = −0.0516, *p*-value = 6.67 × 10^−20^), 3-methyl-2-oxobutyrate (β = −0.0514, *p*-value = 3.82 × 10^−23^), and glycerol-3-phosphate (β = −0.0510, *p*-value = 9.56 × 10^−26^) (Figure 1, Table 1).

MR estimate of the exposure (β_exposure_) represents the causal association between genetically predicted metabolite levels and HbA1c in the whole cohort, while the PRS-metformin interaction (β_G×E_) term reflects modification of this association in metformin users vs. non-users.

In the whole cohort, 1,5-Anhydroglucitol (1,5-AG) showed a protective effect with HbA1c (β_exposure_ = −0.028, *p*-value = 2.7 × 10^−2^), and in metformin users, this protective association was significantly strengthened as indicated by a negative PRS-metformin interaction effect (β_G×E_ = −0.0238, *p*-value 1.39 × 10^−24^).

On the other hand, 3-methyl-2-oxobutyrate (β_exposure_ = 0.040, *p*-value 3.8 × 10^−3^) and glutamate (β_exposure_ = 0.031, *p*-value 1.32 × 10^−2^) showed positive MR effects on HbA1c, showing that genetically higher levels were causally associated with higher HbA1c. However, in metformin users, the negative PRS-metformin interaction effect reversed these causal associations and led to a net protective relationship with HbA1c (β_G×E_ = −0.051, −0.052, *p*-value 3.82 × 10^−23^, 6.67 × 10^−20^, respectively).

Metabolites such as glycine, glutamine, palmitoyl sphingomyelin (d18:1/16:0), 1-ribosyl-imidazoleacetate, and pro-hydroxy-pro demonstrated negative β_exposure_ effects, consistent with protective causal associations with HbA1c among the whole cohort. However, in users, the positive interaction effect attenuated these protective associations, reflecting an increase in their HbA1c% under treatment (Table 2).

## 3. Discussion

In this study, we applied a Gene–Environment interaction Mendelian Randomization (MR-GxE) method to evaluate whether the causal association between genetically influenced variation in circulating metabolites and glycemic control differs according to metformin use. By combining metabolite-specific polygenic risk scores within a modified two-stage least squares model, we identified 22 metabolites for which the genetic effects of the metabolite-HbA1c relationship were significantly modified by metformin exposure. These results demonstrate that metformin changes the metabolic pathways through which genetic predisposition influences glycemic outcomes, highlighting the heterogeneity of treatment responses that are not shown by the conventional MR approach.

Within the MR-G×Eframework, the interaction estimates presented in this study represent the effect modification in the presence of treatment. Specifically, it captures differences in the magnitude or direction of the genetically predicted metabolite-HbA1c association between metformin users and non-users. A positive interaction estimate indicates a stronger genetic causal effect of glycemic response specifically in metformin users, and a negative estimate indicates the attenuation or reversal of causal effects in the presence of metformin. The causal effect of the genetically influenced metabolite levels on HbA1c in the general cohort is explained by the exposure beta effect, which is compared with the interaction term that is specific to metformin users.

In line with this interpretation, three patterns of treatment-dependent effects were observed. Firstly, 1,5-anhydroglucitol showed a negative interaction effect with metformin treatment that strengthened an already protective baseline association, confirming evidence of higher levels of 1,5 AG associated with HbA1c-lowering effect [17,18]. Secondly, metabolites such as glutamate and 3-methyl-2-oxobutyrate, the positive baseline associations were reversed and were negative in the interaction term, showing a net protective association with HbA1c among metformin users. Thirdly, protective baseline associations observed in glycine, glutamine, and palmitoyl sphingomyelin (d18:1/16:0) demonstrated a positive interaction effect, which attenuated the protective association and reflected a reduced HbA1c-lowering effect under metformin treatment. Collectively, these patterns show that metformin use changes both the magnitude and direction of genetically driven metabolite-HbA1c relationships.

The metabolites demonstrating significant PRS-metformin interaction effects gather within metabolic pathways that have well-established links to glucose metabolism, insulin sensitivity, and amino acid pathways that are relevant in metformin’s mechanism of action [6]. Glycine and glutamine–glutamate metabolism have been shown to consistently and negatively correlate with T2D [19], as studies have shown that individuals who are insulin resistant or have impaired glucose metabolism have lower levels of circulating glycine and glutamine [20,21] and elevated glutamate levels [22]. Our previous study also demonstrated that higher glycine and glutamine levels and lower glutamate levels are associated with a good response to metformin among T2D individuals [6]. In this study, while the genetically influenced glycine shows a protective basal association with HbA1c; however, the interaction term shows a strong positive causal effect among metformin users. Given evidence that glycine is negatively associated with metformin use over a period of time [23], the positive interaction for glycine may reflect that the genetically driven association with HbA1c is shifted in a more positive direction among metformin users. In the context of the protective baseline effect, this is consistent with the attenuation of genetically driven glycine-related benefits due to pathway saturation or reduced metabolic variability in long-term users rather than reversal of its role in glycemic regulation.

In contrast, glutamate showed positive basal genetic associations with HbA1c, indicating non-protective effects on glycemic control, while negative PRS-metformin interaction effects attenuated these associations among metformin users, resulting in a more favorable outcome under treatment. This is consistent with established evidence that metformin inhibits glutaminase activity and reduces glutamate accumulation [24,25]. A similar pattern was observed for 3-methyl-2-oxobutyrate, which is an endogenous metabolite and branched-chain keto acid involved in the metabolism of amino acid valine [26]. Metformin can potentially suppress the expression or the activity of enzymes in BCAA catabolism, specifically branched-chain aminotransferase (BCAT2) and branched-chain alpha-keto acid dehydrogenase E1a (BCKDHa) [27]. By inhibiting BCAA catabolism, metformin may aid in the regulation of circulating levels of gluconeogenic substrates like 3-methyl-2-oxobutyrate and thereby contribute to its overall effect in lowering blood glucose production in the liver [28].

A subset of metabolites such as creatinine, gamma glutamylcitrulline, N-acetylthreonine, 3-methyl-2-oxovalerate, glycerol-3-phosphate, and lipid-related metabolites (1-(1-enyl-palmitoyl)-GPC (P-16:0), 1-(1-enyl-palmitoyl)-2-linoleoyl-GPC (P-16:0/18:2), sphingomyelin (d18:1/22:1, d18:2/22:0, d16:1/24:1), fructose, and methyl-glucopyranoside) showed no significance with causal association with HbA1c by itself but demonstrated significant PRS-metformin interaction effects. These metabolites are closely related to insulin resistance [29], hepatic gluconeogenesis [30], and nitrogen metabolism [31], which are all modulated by metformin. These results indicate that genetically driven variation in these metabolites influences glycemic control only in the context of metformin use, highlighting metabolic effects that are conditional on pharmacological treatment and therefore invisible in conventional MR analysis. Notably, we observed both positive and negative interaction estimates, which suggests pathway-specific amplification or attenuation of genetic effects under metformin. These metabolites are related to amino acid turnover [32], gamma-glutamyl cycling, glucose handling [33], lipid membrane remodeling [34], and renal metabolism, and are in line with metformin’s wider effects on mitochondrial function, substrate flux, and cellular energy production [35]. The presence of both positive and negative interaction estimates also supports biological heterogeneity instead of a uniform metabolic response. Some individuals have genetic profiles that may amplify metformin’s glucose-lowering effects, whereas others may counteract or limit its efficacy [36], which backs the inter-individual variability in HbA1c response seen in T2D patients under the same treatment. Thus, our study findings support a model in which metformin unveils or alters genetically mediated metabolic influences on HbA1c rather than acting through baseline metabolite levels alone. This underscores the importance of interaction-based causal approaches for identifying treatment-dependent metabolic determinants of glycemic response.

A major strength of this study is the integration of metabolomics, genetics, and pharmacological exposure in an interaction MR framework that can capture treatment-dependent causal inference. The use of PRS increased the instrument strength and enabled the investigation of metabolites influenced by many genetic variants. However, this study is not completely free from its limitations. Firstly, the statistical power to detect the interaction effect is limited, and some insignificant findings may reflect a lack of power rather than the absence of an effect. Secondly, metformin use was modeled as a binary exposure and does not inform variations in dose, duration or adherence. Third, even though the interaction method removes certain pleiotropic concerns, bias may come if the genetic predisposition influences metformin treatment intensity. Since the study only utilizes the data from Qatar Biobank, replication in independent cohorts and longitudinal studies is essential to confirm the robustness and generalizability of these findings. Moreover, the study is cross-sectional and uses single-time-point metabolomics data; thus, the potential variation due to diet, medication, and physical activity could not be assessed.

Nonetheless, our emerging data has revealed metabolites whose genetically driven associations with HbA1c were strengthened, attenuated, or reversed in the presence of metformin, as well as metabolites with no baseline causal effect showing relevance only under treatment exposure. This study demonstrates that genetic influences on metabolites and their relationship to HbA1c can possibly be modified by metformin treatment. In conclusion, this work underlines the importance of adding pharmacological context into causal inference analyses and provides a foundation for further studies aimed at shaping precision treatment for type 2 diabetes.

## 4. Materials and Methods

### 4.1. Study Cohort

**Figure 2:**
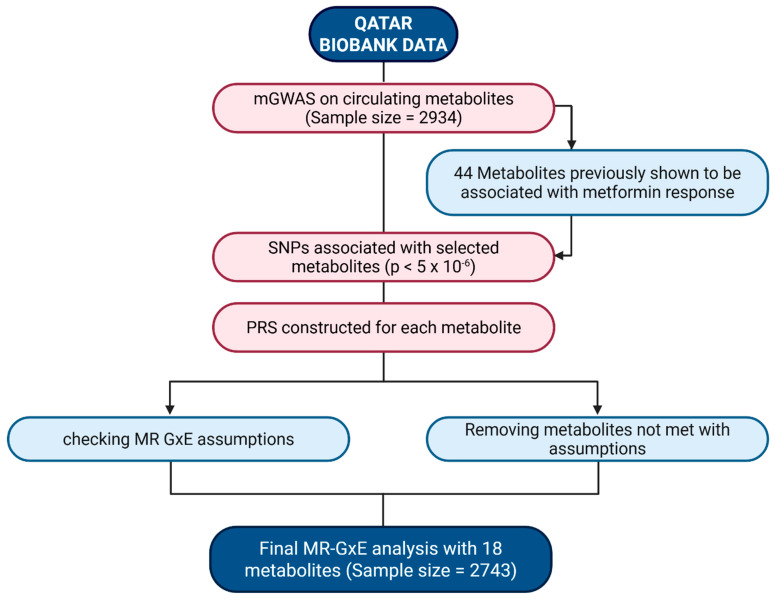
Summary of the study design and analytical workflow for the MR-G×Eanalysis.

Study participants were recruited from Qatar Biobank (QBB), a population-based cohort of native Qataris and long-term residents of ≥15 years in Qatar [37]. Prior to data and sample collection, all participants provided informed consent. As part of recruitment, participants were asked to complete a standardized questionnaire, self-reporting information including history of diseases, medication intake, and diet. In addition, clinical measurements and biological samples, including blood, were obtained from all participants. This study comprises 2934 participants who had complete data on age, BMI, sex, and metabolomics profile. This study was approved by the Institutional Review Boards of QBB (E-2024-QF-QBB-RES-ACC-00199-0267) and Qatar University (QU-IRB 215/2024-EM). A summary of the study design and the analytical workflow is depicted in Figure 2.

### 4.2. Metabolomics

Serum samples from QBB (*n* = 2934) participants were collected and untargeted metabolomics analysis was performed at the Anti-Doping Lab, Qatar, using the Metabolon platform. The protocol used for the analysis was described in detail previously [38]. Briefly, metabolic profiling was achieved using a Waters ACQUITY ultra-performance liquid chromatography (UPLC) system (Waters Corporation, Milford, MA, USA) along with a ThermoScientific Q-Exactive high-resolution/accurate mass spectrometer (Thermo Fisher Scientific, Inc., Waltham, MA, USA). This mass spectrometer (MS) was provided with a heated electrospray ionization (HESI-II) source along with an Orbitrap mass analyzer, which operates at 35,000 mass resolution. Protein content was removed from the serum samples using methanol and the remaining extract was further divided into five fractions to perform comprehensive metabolite profiling using advanced ionization and chromatography methods. Extraction of the raw data, peak identification and quality control processes were performed using Metabolon’s built-in software (Metabolon Inc., Durham, NC, USA). The identification of compounds was performed by comparing them to the libraries, which contain 3300 pure standard compounds. To confirm accurate identification of compounds, the resulting library that matched each compound was examined carefully and corrected if necessary.

### 4.3. Whole-Genome Sequencing of QBB Participants

Whole-genome sequencing (WGS) was performed at Qatar Precision Health Institute (QPHI) as described previously [39]. Briefly, peripheral blood was used to extract DNA, and genomic libraries were prepared using the Qiagen MIDI kit (Qiagen, Hilden, Germany). The genomic libraries were sequenced at the Sidra Medicine facility using the Illumina (San Diego, CA, USA) HiSeq X Ten platform with a desirable coverage of 30×. The quality of fastq files was checked using FastQC (https://www.bioinformatics.babraham.ac.uk/projects/fastqc/, accessed on 12 May 2026), and bwa.kit (v0.7.12) was used to align reads against the human reference genome GRCh38. Picard (v1.117) was used to assess the mapping quality, and gVCF files were generated using GATK 3.4 best practices. Non-autosomal variants, variants with MAF < 5%, Hardy–Weinberg *p*-value < 10^−6^, and genotyping call rate of <90% were filtered, and the remaining 6,375,079 variants and 2743 individuals were used for the final analysis. Variants were also pruned with an LD threshold of r^2^ = 0.5 to include independent variants for the estimation of principal components (PCs) and the relationship matrix. All the QC steps were performed using PLINK-1.9.

### 4.4. Gene x Environment Interaction Analysis and Instrumental Variable Selection

The Mendelian Randomization Gene–Environment (MR-GxE) interaction approach was used in this study. Since the data was used from the same population, a two-stage least squares regression (2SLSR), the *ivreg* method was applied. The formula used in this analysis was adopted from a previously published study of MR-G×E [16]. Our previous study has shown 44 metabolites associated with HbA1c in T2D patients treated with metformin [6]. The primary focus of this analysis was to investigate the potential causal relationships between SNP-associated metabolites, considering metabolite levels as the exposure and HbA1c as the outcome in metformin users and non-users (binary).

SNP-associated metabolites were collectively used to generate a polygenic risk score (PRS) as an instrumental variable (IV). SNPs associated with each metabolite at $p<5\times 10^{-6}$ were used to construct the PRS. The metabolite-associated SNPs were clumped using an $r^{2}$ threshold of 0.001 within a 500 kb window. The clumped SNPs were further evaluated for evidence of horizontal pleiotropy by testing whether they were associated with HbA1c, and confounders (age, sex, or BMI) at p<0.05, and whether they had been reported in public databases such as GWAS Catalog or HuGeAMP as being associated with HbA1c. SNPs that showed association with HbA1c or confounders were subsequently removed. The final list of SNPs was used to calculate PRS for each metabolite using the “sum” function of PLINK [40].

To satisfy the MR-G×Eassumptions, the constructed PRS (IV) was regressed against the metabolite (exposure) and F-statistics were observed to check the strength of IVs. The PRS with F-statistics > 10 was retained. Also, if the PRS (IV) showed an association (*p* < 0.05) with HbA1c, the PRS was excluded. Additionally, to rule out collider bias, only PRS which does not show an association (*p* < 0.05) with metformin use were considered for MR-G×Eanalysis (Supplementary Table S1).

Leave-one-out (LOO) sensitivity analysis was also performed using R (version 4.2.1), at later stages of the analysis, to check the robustness of the results and horizontal pleiotropy.

### 4.5. MR-G×EInteraction Analysis Formula

The product of PRS and metformin users/metformin non-users (binary) was taken as a Gene–Environment (G×E) interaction variable for the analysis, as shown in the formula:

$$ivreg\left(Outcome\;\sim \;Exposure+G\times E+Covariates\;\right|\;PRS+G\times E+\;Covariates\;)$$

Here, outcome = HbA1c, Exposure = metabolite values, PRS = PRS associated with metabolites, G×E = PRS × Metformin-Use (binary), and Covariates = age, gender, BMI, and ancestral PCs 1, 2, 3, and 4.

## 5. Conclusions

Our study showed that the genetic effects on metabolic intermediates are not static but are pharmacologically conditional. Identifying that the association of genetically influenced metabolites with HbA1c is affected by metformin treatment suggests that the genetic influence over metabolic mediators and clinical outcomes can be affected by treatment. This approach, when validated on a larger and independent cohort, can inform stratified treatment plans and identify individuals who are more likely to benefit from metformin based on their genetically influenced metabolic state. Moreover, these metabolic pathways can potentially be secondary targets for intervention, either to improve metformin efficacy or underline treatment resistance.

## Figures and Tables

**Table 1:** Mendelian Randomization Gene–Environment (MR-GxE) interaction results for metabolite-specific polygenic risk scores. Estimates represent the interaction effect between PRS and metformin use, adjusted for age, sex, BMI, and genetic principal components (1–4).

Metabolite	Estimate (β_G×E_)	SE	*p*-Value
N-acetylthreonine	0.030	0.003	2.22 × 10^−29^
1,5-Anhydroglucitol	−0.024	0.002	1.39 × 10^−24^
3-methyl-2-oxobutyrate	−0.051	0.005	3.82 × 10^−23^
Glutamate	−0.052	0.006	6.67 × 10^−20^
Gamma-glutamylcitrulline	0.039	0.004	8.12 × 10^−19^
Fructose	0.023	0.003	2.04 × 10^−17^
Glutamine	0.124	0.022	1.62 × 10^−8^
Palmitoyl sphingomyelin (d18:1/16:0)	0.118	0.024	5.34 × 10^−6^
1-ribosyl-imidazoleacetate	0.058	0.005	1.27 × 10^−26^
Pro-hydroxy-pro	0.034	0.003	1.99 × 10^−23^
Methyl glucopyranoside (alpha + beta)	−0.028	0.003	6.27 × 10^−25^
Glycerol-3-phosphate	−0.051	0.005	9.56 × 10^−26^
Glycine	0.175	0.017	1.48 × 10^−25^
1-(1-enyl-palmitoyl)-2-linoleoyl-GPC (P-16:0/18:2)	0.037	0.003	7.77 × 10^−29^
3-methyl-2-oxovalerate	0.027	0.003	1.17 × 10^−24^
Sphingomyelin (d18:1/22:1, d18:2/22:0, d16:1/24:1)	−0.017	0.002	5.33 × 10^−27^
1-(1-enyl-palmitoyl)-GPC (P-16:0)	−0.025	0.002	1.01 × 10^−25^
Creatinine	−0.027	0.002	7.54 × 10^−30^

**Table 2:** MR-G×Einteraction (left) and exposure effects (right) of metabolite-specific polygenic risk scores on HbA1c. Estimates (β), standard errors (SEs), and two-sided *p*-values are shown. All models were adjusted for age, sex, BMI, and genetic principal components.

	PRS-Metformin Interaction			Exposure (Metabolite)		
Metabolite	β_G×E_	SE	*p*-Value	β_exposure_	SE	*p*-Value
1,5-Anhydroglucitol	−0.024	0.002	1.39 × 10^−24^	−0.028	0.012	2.70 × 10^−2^
3-methyl-2-oxobutyrate	−0.051	0.005	3.82 × 10^−23^	0.040	0.014	3.80 × 10^−3^
Glutamate	−0.052	0.006	6.67 × 10^−20^	0.031	0.012	1.32 × 10^−2^
Glutamine	0.124	0.022	1.62 × 10^−8^	−0.085	0.016	5.51 × 10^−8^
Palmitoyl sphingomyelin (d18:1/16:0)	0.118	0.024	5.34 × 10^−7^	−0.044	0.015	3.82 × 10^−3^
1-ribosyl-imidazoleacetate	0.058	0.005	1.27 × 10^−26^	−0.076	0.034	2.75 × 10^−2^
Pro-hydroxy-pro	0.034	0.003	1.99 × 10^−23^	−0.066	0.030	2.93 × 10^−2^
Glycine	0.175	0.017	1.48 × 10^−25^	−0.304	0.070	1.61 × 10^−5^

## Data Availability

The original contributions presented in this study are included in the article/Supplementary Materials. Further inquiries can be directed to the corresponding author.

## Supplementary Materials

The following supporting information can be downloaded at: https://www.mdpi.com/article/10.3390/ph19050780/s1, Table S1: MR_Interaction_final.
